# Integrating partner notification and contact tracing services across Europe: findings from the Integrate project

**DOI:** 10.1186/s12879-021-06445-5

**Published:** 2021-09-13

**Authors:** Shannon Glaspy, Lella Cosmaro, Chryssoula Botsi, Maria Stamou, Maria Giannopoulou, Aikaterini M. Isari, Cheyenne Downey, Tina Mc Hugh, Gordana Avramovic, John S. Lambert

**Affiliations:** 1 School of Medicine, University College Dublin, Dublin, Ireland; 2 Fondazione LILA Milano, Italian League for Fighting AIDS, Milan, Italy; 3 National Public Health Organization (NPHO)/EODY, Marousi, Greece; 4 Unit of Infectious Diseases Andreas Syggros Hospital, Athens, Greece; 5 Infectious Diseases Department, Mater Misericordiae University Hospital, Dublin, Ireland

**Keywords:** Partner notification, Contact tracing, Integration, Barriers

## Abstract

**Background:**

Partner notification/contact tracing (PN/CT) is a process whereby people diagnosed with an infectious disease notify their sexual and needle-sharing partners/close contacts and invite them for testing and treatment due to exposure to the disease. PN is a necessary testing and prevention tool supported by the European Centre for Disease Prevention and Control (ECDC) and World Health Organization (WHO). Traditionally, PN efforts have been siloed within disease areas, with separate pathways and systems responsible for specific diseases. The INTEGRATE project sought to improve PN/CT outcomes by sharing knowledge across diseases and countries.

**Methods:**

INTEGRATE used two mapping exercises to assess the PN landscape in Europe and identify areas for integration and cross-learnings for Sexually Transmitted Infections (STIs) and Tuberculosis. Mapping exercises were surveys to 29 consortium partners and in-depth qualitative interviews at four selected pilot sites: Ireland, Greece, Romania and Italy.

**Results:**

Areas for the improvement of PN/CT emerged: lack of resources and insufficient staff training, different modes of disease transmission, country-specific laws and regulations, the advent of General Data Protection Regulation (GDPR), differences in healthcare system pathways, historical concerns, and cultural differences. Activities highlighted key areas PN/CT outcomes could be improved, including PN/CT specific trainings for staff, improving knowledge on laws, regulations, guidelines and pathways and creating a country/region specific Standard Operating Procedures (SOPs) for PN/CT, incorporating information on all four disease areas. Findings were analyzed and three key areas were identified and implemented for knowledge transfer namely the creation of an online repository of European country guidelines, the transfer of SOPs and PN training in pilot sites.

**Conclusion:**

A major finding of the project was challenges associated with incorporating Tuberculosis (TB) contact tracing alongside other infectious diseases. Professionals in the field, emphasized that integrating TB contact tracing with the other disease areas would be challenging and arguably unjustified, due to the different ways of transmission of TB and because well-established historical pathways for TB in public health systems already exist. However, the success of TB services presents an ideal model to draw from when strengthening PN systems for other infectious diseases.

## Background

Partner notification (PN)/contact tracing (CT) is a voluntary process in which people diagnosed with an infectious disease inform their sexual and needle-sharing partners/close contacts and invite them for testing and treatment. Patients refer on their own (patient referral) or they can be assisted by healthcare providers or peers (healthcare-assisted partner notification) or by a healthcare worker (provider referral) where regulations allow. It is an essential testing and secondary prevention tool recommended by the ECDC and WHO [1, 2], The WHO notes that voluntary assisted partner notification should be offered as a comprehensive package of testing and treatment [1], and other bodies such as the ECDC and the International Union against Sexually Transmitted Infections (IUSTI), support the inclusion of partner notification practices as an essential component of sexual healthcare [2, 3]. Tuberculosis contacts should be investigated systematically and actively for TB infection and disease. Such interventions are called ‘tuberculosis contact investigations’. They contribute to early identification of active TB, thus decreasing its severity and reducing transmission of *Mycobacterium tuberculosis* to others, and identification of latent TB infection (LTBI), to allow preventive measures [4, 5].

Partner notification not only helps control the spread of STIs and reduces STI related morbidity and mortality but is also cost-effective in reaching those who are asymptomatic. The ECDC notes the cost-effectiveness of PN/CT is due to high positivity rates among people contacted and tested [2]. More specifically, Public Health England reported a 3.9% new HIV diagnosis in partners brought for testing in 2018 [6].

There are many forms of partner notification, none of which have been shown to be universally preferred or approved to be more effective than the others [1, 2]. Patients may prefer a personal conversation with their partners [7], healthcare-assisted partner notification or anonymous PN through the use of new Information and Communication Technology (ICT) tools [1]. Additionally, there are no common EU/EEA guidelines on how to perform TB contact tracing or partner notification for HIV, STIs and viral hepatitis [2] that all EU member states subscribe to and follow. Many member states do not have national guidelines for TB contact tracing, yet of those that do exist, the criteria for selecting contacts, the screening process and the prescription of preventative treatment differs [8]. International and European bodies, such as WHO, ECDC, IUSTI, ERS and more provide guidance on what should be included in national guidelines, but there is no standard European guideline for any of the four disease areas, leaving staff at testing and healthcare services responsible for creating their own PN pathways and procedures.

The ECDC has called for research to determine the most effective approaches to PN in Europe, to harmonize European wide recommendations on PN, develop common indicators for PN and to improve awareness to the importance of PN among policy-makers [2]. Previously, partner notification/contact tracing attempts have been neglected within disease areas as individual pathways and systems cater for specific infectious disease separately.

INTEGRATE is a European Commission co-funded Joint Action (2017–2021) and the first European project on the integration of infectious diseases in testing services, data collection and country responses [9]. Gathering 29 partners across Europe, it aims to combine partner notification and contact tracing experiences and best-practice and to enhance partner notification in high-risk populations. The aim was to organise some knowledge sharing and transfer between participating countries.

## Methods

National Ministries of health in 16 European Union (EU) and neighbouring countries nominated 29 organisations (from non-governmental institutions (NGOs) to public health institutes and hospitals), to form a consortium for the INTEGRATE project. For the partner notification component of INTEGRATE, four pilot countries (Ireland, Romania, Greece and Italy) were selected as they ensured regional representation across Europe minimizing geographical biases amongst responsive organisations. Greece was selected as a country where partner notification is not compulsory, but routinely carried out for at least one STI. Greece experienced a high HIV outbreak in 2011–2013 which is now under control [10]. It was considered to provide regional representation for the South of Europe. Romania was selected as a country where partner notification is compulsory and routinely carried out for at least one STI. Romania has a high prevalence of Hepatitis C and is also a high priority country for TB [11, 12]. It was considered to provide regional representation for the east of Europe. Italy was selected as a country where partner notification is compulsory and routinely carried out for at least one STI [13]. HIV rates almost doubled since 2008 with a large proportion of late presenters [14]. It was considered to provide regional representation for the West of Europe. Ireland was selected as a country where partner notification is not compulsory, but routinely carried out for at least one STI. Almost half of registered PLHIV were born abroad in Ireland [15] and there is a high burden of Hepatitis amongst migrant population [16]. It was considered to provide regional representation for the North of Europe. Leadership was held by the Dublin site -University College Dublin- for the interventions reported in this article.

Two types of interventions were carried out. The first intervention was a mapping exercise. It included a baseline survey of the 29 INTEGRATE partner organizations from 16 different countries, to gain an understanding of the current situation of PN/CT services across Europe. The survey was circulated among all INTEGRATE partners and collaborating stakeholders in November–December 2017 via an online system (RedCap). The survey questions covered current practices, legal requirements, reporting, challenges, guidelines and responsibilities. Participating stakeholders were varied in nature, including clinical, educational, civil society and public health organizations selected to be part of the consortium.

This was followed by a second mapping exercise consisting of qualitative interviews conducted in the 4 chosen pilot countries (Ireland, Greece, Italy, Romania) conducted with key stakeholders who opted to participate. Stakeholders were interviewed for each disease area to gain a thorough understanding of the PN/CT pathways. A semi-structured interview guide was translated and used for all interviews, with responses recorded and translated to English. Sites were interviewed about pathways, barriers, monitoring, laws and guidelines to gain an understanding of how partner notification pathways operate in day-to-day life for HIV, STI’s, Hepatitis and TB. Moreover, they were asked to produce a flowchart for all diseases and to consider potential disease areas that could be integrated for PN/CT. Results were analyzed and coded by the Irish site highlighting areas to be considered for integration from a disease to another and possibilities for knowledge transfer from a country to another.

After the mapping exercises, key areas were highlighted where knowledge transfer could be considered between pilot countries. Due to a limited capacity under this joint action, three key knowledge areas were selected for an implementation of knowledge transfer to all member states, in participating countries and at pilot sites.

## Results

### Mapping exercise 1- baseline survey

The survey reported on country requirements for PN/CT. Table 1 illustrates responses that were given by interviewees when asked if PN/CT is mandatory by disease area in their country. Mandatory PN/CT reporting was reported as necessary by six to nine stakeholders out of the 27 who participated for HIV, HBV, HCV, chlamydia, syphilis and gonorrhoea. For TB, 15 out of the 27 partners reported mandatory PN/CT. Table 2 shows the answers to whether reporting of PN/CT is legally required by disease area. For HIV, HBV, HCV, chlamydia, syphilis and gonorrhoea stakeholders reported that they were legally required to do PN/CT in 3 to 6 cases. For TB, it was reported that PN/CT was legally required in 10 out of 27 cases. Table 3 shows responses for whether or not there are guidelines for PN/CT by disease area. Guidelines were mostly present for TB with 13 out of 27 stakeholders and least present for HCV with 5 out 27 stakeholders. Tables also present conflicting and unknown answers, or where there was simply no information given. Portugal, Greece, Lithuania, Romania, Hungary and Croatia, Estonia, Poland and Spain gave conflicting replies or did not know or answer in one or two dimensions identified in Tables 1, 2 and 3.

**Table 1:** Is PN/CT mandatory in your country?

***Country***	***Organisation type***	***HIV***	***HBV***	***HCV***	***Syph***	***Chla***	***Gonorr***	***TB***
Ireland	University	No	No	No	No	No	No	Yes
Italy	NGO	No	No	No	No	No	No	Yes
Italy	NGO	No	No	No	No	No	No	No
Poland	Public Health	No	No	No	No	No	No	No
Poland	University	No	No	No	Yes	No	No	No
Croatia	NGO	No	No	No	No	No	No	No
Croatia	Public Health	Yes	Yes	Yes	Yes	–	–	Yes
Croatia	NGO	No	No	No	No	No	No	–
Croatia	NGO	No	–	–	–	–	–	–
Slovakia	University	No	Yes	Yes	Yes	Yes	Yes	Yes
Hungary	University	No	–	–	Yes	No	Yes	–
Hungary	Public Health	Yes	Yes	No	yes	Yes	Yes	Yes
Lithuania	Hospital	No	No	No	No	No	No	–
Lithuania	Hospital	Yes	–	–	yes	yes	yes	yes
Lithuania	Public Health	Yes	Yes	Yes	Yes	Yes	Yes	Yes
Lithuania	Public Health	No	No	No	No	No	No	No
Spain	Public Health	No	–	–	–	–	–	yes
Malta	Public Health	Yes	Yes	Yes	Yes	Yes	Yes	–
Romania	Hospital	Yes	–	–	–	–	–	Yes
Romania	Hospital	Yes	–	–	–	–	–	Yes
Slovenia	Public Health	No	No	No	No	No	No	Yes
Estonia	Public Health/Research	Yes	Yes	Yes	Yes	No	No	Yes
Greece	Public Health	–	–	–	–	–	–	Yes
Greece	Research Institute	–	–	–	–	–	–	–
Serbia	Public Health	Yes	Yes	Yes	Yes	Yes	Yes	Yes
Portugal	NGO	No	No	No	No	No	No	Yes
Norway	Public Health	–	–	–	–	–	–	–
**Mandatory reporting**		9/27	7/27	6/27	10/27	6/27	7/27	15/27

*NGO Non-Governmental Organisation, *HIV* Human Immunodeficiency Virus, *HBV* Hepatitis B Virus, *HCV* Hepatitis C Virus, *Syph* Syphilis, *Chla* Chlamydia, *Gonorr* Gonorrhea, *TB* Tuberculosis

**Table 2:** Is reporting on PN/CT legally required in your country?

***Country***	***Organisation type***	***HIV***	***HBV***	***HCV***	***Syph***	***Chla***	***Gonorr***	***TB***
Ireland	University	No	No	No	No	No	No	Yes
Italy	NGO	No	No	No	No	No	No	No
Italy	NGO	No	No	–	Yes	No	No	No
Poland	Public Health	No	No	No	No	No	No	No
Poland	University	No	No	No	No	No	No	–
Croatia	NGO	–	–	–	–	–	–	–
Croatia	Public Health	No	No	No	No	No	No	No
Croatia	NGO	No	Yes	Yes	Yes	Yes	Yes	Yes
Croatia	NGO	No	–	–	Yes	–	Yes	–
Slovakia	University	Yes	Yes	No	Yes	Yes	Yes	Yes
Hungary	University	No	No	No	No	No	No	No
Hungary	Public Health	Yes	–	–	yes	Yes	Yes	Yes
Lithuania	Hospital	No	No	No	No	No	No	No
Lithuania	Hospital	Yes	Yes	Yes	yes	yes	yes	yes
Lithuania	Public Health	Yes	–	–	–	–	–	Yes
Lithuania	Public Health	Yes	–	–	–	–	–	Yes
Spain	Public Health	No	No	No	No	No	No	yes
Malta	Public Health	–	–	–	–	–	–	–
Romania	Hospital	No	No	No	No	No	No	No
Romania	Hospital	No	No	No	No	No	No	No
Slovenia	Public Health	No	No	No	No	No	No	–
Estonia	Public Health/Research	Yes	Yes	Yes	–	–	–	Yes
Greece	Public Health	No	No	No	No	No	No	Yes
Greece	Research Institute	–	–	–	–	–	–	–
Serbia	Public Health	No	No	No	No	No	No	No
Portugal	NGO	–	–	–	–	–	–	–
Norway	Public Health	–	–	–	–	–	–	–
**Legally required reporting**		6/27	4/27	3/27	6/27	4/27	5/27	10/27

*NGO Non-Governmental Organisation, *HIV* Human Immunodeficiency Virus, *HBV* Hepatitis B Virus, *HCV* Hepatitis C Virus, *Syph* Syphilis, *Chla* Chlamydia, *Gonorr* Gonorrhea, *TB* Tuberculosis

**Table 3:** Are there any guidelines for PN/CT in your country?

***Country***	***Organisation type***	***HIV***	***HBV***	***HCV***	***Syph***	***Chla***	***Gonorr***	***TB***
Ireland	University	No	No	No	No	No	No	Yes
Italy	NGO	–	–	–	–	–	–	–
Italy	NGO	No	No	No	No	No	No	Yes
Poland	Public Health	No	No	No	No	No	No	No
Poland	University	No	No	No	No	No	No	No
Croatia	NGO	Yes	Yes	Yes	Yes	Yes	Yes	Yes
Croatia	Public Health	Yes	Yes	Yes	Yes	Yes	Yes	Yes
Croatia	NGO	–	–	–	–	–	–	–
Croatia	NGO	No	–	–	–	–	–	–
Slovakia	University	No	Yes	Yes	Yes	Yes	Yes	Yes
Hungary	University	No	–	–	Yes	–	Yes	–
Hungary	Public Health	–	–	–	–	–	–	–
Lithuania	Hospital	–	–	–	–	–	–	Yes
Lithuania	Hospital	Yes	–	–	–	–	–	Yes
Lithuania	Public Health	–	–	–	–	–	–	–
Lithuania	Public Health	Yes	–	–	Yes	Yes	Yes	Yes
Spain	Public Health	Yes	–	–	Yes	Yes	Yes	Yes
Malta	Public Health	No	No	No	No	No	No	No
Romania	Hospital	Yes	–	–	Yes	Yes	Yes	Yes
Romania	Hospital	Yes	Yes	Yes	Yes	Yes	Yes	Yes
Slovenia	Public Health	No	No	No	No	No	No	–
Estonia	Public Health/Research	Yes	Yes	Yes	Yes	Yes	Yes	Yes
Greece	Public Health	Yes	No	No	–	–	–	Yes
Greece	Research Institute	–	–	–	–	–	–	–
Serbia	Public Health	No	No	No	No	No	No	Yes
Portugal	NGO	–	–	–	–	–	–	–
Norway	Public Health	–	–	–	–	–	–	–
**Reported guidelines**		9/27	5/27	5/27	9/27	8/27	9/27	13/27

*NGO Non-Governmental Organisation, *HIV* Human Immunodeficiency Virus, *HBV* Hepatitis B Virus, *HCV* Hepatitis C Virus, *Syph* Syphilis, *Chla* Chlamydia, *Gonorr* Gonorrhea, *TB* Tuberculosis

### Mapping exercise 2- qualitative interviews at pilot sites

While some findings are country and/or disease area specific, there were many findings that were echoed across settings and disease areas.

A first key theme was a lack of resources and time to ensure that partner notification is handled appropriately and effectively. Another theme was confusion among interviewees in respect to certain aspects of PN/CT services, including the official legal requirements and available guidelines, as well as knowledge of how PN/CT is conducted on the ground and which staff member has the responsibility to oversee and conduct it. Some stakeholders noted that this confusion is compounded by a lack of uniform set-up for partner notification across countries and healthcare systems. In Ireland, each of the local departments of health act independently, forming their own pathways and methods for partner notification. In Italy, some regions have created their own guidelines for partner notification, while others may utilize international guidelines or not specify any at all.

Across sites and disease areas a key theme emerged regarding the need for increased training on partner notification to improve service outcomes. The interviewed stakeholders noted that healthcare workers need training on PN/CT issues and the regulatory framework that surround it in their specific context, to feel confident in conducting these services and counselling patients on the topic. In Greece, for example, a law (2472/1997) that protected patient confidentiality made healthcare workers feel less confident in what aspects of partner notification they were legally allowed to counsel and assist patients. This law has since been replaced by the EU General Data Protection Regulation (GDPR) 2016/679, resulting in similar effects in Greece and all EU countries. Across Europe, the advent of the GDPR in May 2018 led to concerns related to patient confidentiality within the PN/CT process. Clarification on the laws that surround the PN/CT process is needed. In the instance that there are national or regional guidelines for PN/CT, stakeholders interviewed reflected that the healthcare workers that conduct PN/CT services do not have access to them or are not aware of them, rendering them ineffective. Training on how to conduct PN/CT services could be applied to numerous testing and counselling settings, especially using country specific guidelines and information.

The interviews highlighted that monitoring PN/CT remains a challenge. Not only do confidentiality laws and requests for anonymity make it difficult to audit the effectiveness of PN, but there are also difficulties surrounding data protection with new GDPR requirements.

Additionally, a national registry of PN/CT is not feasible in countries without a national registry for infectious diseases notification. Furthermore, it was widely acknowledged that auditing PN/CT outcomes could improve resources and support for PN/CT services.

The key theme emerging regarding potential integration across diseases was that while HIV, STIs and in some instances viral hepatitis could be integrated without significant systematic changes in current operations, TB contact tracing is vastly different. In most settings, TB care and the contact tracing that accompanies it have pathways set in institutions which are separate from the other disease areas. More specifically, HIV, STIs and viral hepatitis are often housed within infectious diseases, whilst TB tends to be housed in respiratory medicine or public health. Furthermore, as the mode of transmission for TB is significantly different than that of the other disease areas, being an airborne disease spreading through inhalation of droplets by close contacts, it follows that the contact tracing efforts for TB must be structured in a different manner. This factor also affects the indicators that would be used to determine the effectiveness of PN/CT efforts. Thus, a common list of indicators could work for HIV, STIs and potentially hepatitis, but these would not all be applicable to TB.

Following the online survey and the qualitative interviews, key areas were considered by the consortium for integration across disease areas and for knowledge and expertise transfer between sites.

### Knowledge transfer 1- creation of an online repository of PN documents available to member states

One of the conclusions drawn from the mapping exercises was that there are no common EU/EEA guidelines on how to perform TB contact tracing or partner notification for HIV, STIs and viral hepatitis. After review of the mapping results the consortium decided to create a repository displaying national contact tracing and partner notification guidelines or the specific sections of these guidelines that were applicable for the selected disease area as well as any relevant laws of several European countries. National guidelines and documentation were collected from each country, then translated and included in a public online repository of documents. Both the original and the English versions were made available to the public on the INTEGRATE website to facilitate the availability of a knowledge base [2]. Table 4 shows the country specific guidelines which were collected during the joint action.

**Table 4:** Country specific guidelines collected by Integrate^*^

Country	HIV/STIs	Viral Hepatitis	TB
Croatia	No	No	Yes
Denmark	Yes	No	Yes
Greece	Yes	Yes	Yes
Hungary	Yes	No	Yes
Ireland	No	Yes	Yes
Italy	No	No	Yes- Regional
Lithuania	Yes	No	Yes
Poland	No	No	No
Portugal	No	No	No
Romania	No	No	Yes
Serbia	Yes	Yes	Yes
Slovakia	Yes	No	No
UK	Yes	No	Yes
**Total**	7/13	3/13	10/13

*All guidelines are accessible at: https://integrateja.eu/content/partner-notification-guidelines

^*^HIV Human Immunodeficiency Virus, *STI* Sexually Transmitted Infections, *TB* Tuberculosis

### Knowledge transfer 2- creation of standard operating procedures for pilot sites

To address barriers identified in the mapping exercises and to support healthcare staff in conducting PN/CT, in November 2018 partners involved in improving PN services in the pilot countries decided to transfer knowledge regarding Standard Operative Procedures. A possibility to share knowledge from Ireland with other pilot countries was identified as SOPs were in place in Ireland. They were translated and shared with Italian and Greek partners. An Italian Infectious Disease specialist’s expert input was sought to advise on adapting Irish SOPs to an Italian context. The proposed SOPs were presented to the Ministry of Health during a National Stakeholder Meeting [Ref supplement article # 10]. Standard Operating Procedures (SOPs) provide the health professional the appropriate knowledge to manage PN/CT in order to address the needs of early diagnosis and an effective secondary prevention setting quality standards. The SOPs describe the procedure and define pathways and algorithms.

### Knowledge transfer 3- training in PN

The qualitative mapping exercise identified a need for training. Partner notification training existed and had been implemented at the Irish site since 2006 under a training module specifically focusing on PN developed under the Sexually Transmitted Infections Foundation (STIF) course of the British Association for Sexual Health and HIV (BASHH). The training was adapted and permissions were sought from BASHH to pilot it in Italy. The training was piloted in September 2019 where two facilitators (partners in INTEGRATE), after being trained according to the PN STIF Facilitators Manual, offered a 2-day residential course on PN attended by 31 community health workers. The pilot training received a very positive evaluation from participants, who indicated that they gained good competence on relevant PN procedures and on legal requirements/restrictions brought about by the GDPR**.**

## Discussion

### Key findings

Many member states have national guidelines for TB contact tracing, yet of those that do exist the criteria for selecting contacts, the screening process and the prescription of preventative treatment differs [17, 18]. As demonstrated in the ECDC report [2] some countries make partner notification mandatory, while others have no requirements to engage in partner notification services; some allow healthcare providers to assist patients in notifying partners while others only allow patient referral. This variation can be a source of great concern to healthcare providers who must balance legal requirements and patient confidentiality, with a duty of care to partners. International bodies, such as WHO, ECDC, IUSTI and more provide guidance on what should be included in national guidelines, but there is no standard European guideline for any of the four disease areas that all EU countries follow. Specialty groups within the EU, for example those focused on TB have established standards, but these do not exist for all infectious diseases [19].

The partner survey responses demonstrated significant levels of confusion and uncertainty surrounding the partner notification/contact tracing laws, regulations and practices in each country. Often respondents could outline general principles or describe how PN/CT services were carried out yet could not identify any clear systems or pathways. These responses point to a lack of knowledge/understanding of how or if PN/CT is conducted within these contexts and under which regulations and procedures. Many participants noted that often guidelines for partner notification for HIV and STIs are combined, but not for hepatitis and TB. Despite a lack of in-depth knowledge of how partner notification/CT occurs, respondents were able to identify numerous barriers and challenges that impede the process. Such as, limited resources, limited time, limited staff, lack of clear guidelines, lack of patient education, confidentiality issues and stigma [20]. Additionally, respondents noted that in order to improve PN/CT efforts, there was a need for more training of healthcare providers and community-testing staff, introducing national guidelines on PN/CT and sharing experiences of PN/CT with other countries. Of all the disease areas, responses to TB contact tracing appeared to be the most comprehensive and best understood, with participants often identifying guidelines, documents or outlining the national procedures involved in TB contact tracing. The partner survey responses and mapping exercise indicated that TB contact tracing appears to be the most comprehensive and best resourced of all the disease areas, with many countries demonstrating well established pathways for TB contact tracing. The history of TB in Europe led to significant resources and well-established protocols, many of which still remain today. Recent literature also demonstrates that the majority of EU countries have a defined TB control structure with central management and/or national guidelines [19].

In the baseline survey and the mapping exercise, INTEGRATE partners noted that knowledge transfer could be done via the introduction of PN training and access to guidelines and SOPs, and could greatly improve partner notification outcomes with minimal efforts. Training can increase staff knowledge of PN practices, options and ways to support patients, giving staff the confidence to encourage patients through partner notification. For healthcare providers to support patients in partner notification, it is important that they understand the applicable national/EU laws. This was successfully implemented in the project but within a limited number of sites.

### Limitations

It is important to note that mapping survey findings do not indicate that PN/ CT does not occur, particularly as the respondents could be answering from the point of view of their particular organization (which may not be a clinical/public health organization involved with PN/CT) rather than reflecting national level operating procedures. Moreover, INTEGRATE only had a limited capacity to operate knowledge transfer between partner sites once knowledge transfer areas were identified.

### Implications for practice and policy

The legal environment surrounding partner notification varies greatly from country to country [20], creating a significant barrier for cross-border training programs and guidelines [13]. While a number of countries do not have specific laws that prohibit or mandate PN, some have conflicting laws. For example, Hungary has a law that requires patients to give information on their partners for specific STIs, yet these instructions are in conflict with laws that protect patient confidentiality and anonymity [13]. In Greece and Italy, patient confidentiality laws protect against mandatory or involuntary PN, yet there are provisions for a doctor to seek permission to disclose a patient’s status against their will in rare cases once it is in the best interest of the patient and/or society [21, 22]. Despite the effectiveness of PN/CT in finding new infections, some countries still have laws which act as a barrier to this process, by criminalizing transmission of disease and increasing stigma [20]. When discussing the potential adverse outcomes of partner notification, it is important to highlight that partner notification is not mandatory and should always be considered in light of each patient’s situation (as noted by WHO, UNAIDS, ECDC, IUSTI, etc.) [23]. Partner notification should be supported as a voluntary process, ensuring the patient’s safety [24].

A final area of concern centres around, the introduction of the General Data Protection Regulation (GDPR) 2016/679 which regulates data protection and privacy in the EU and EEA. GDPR was implemented on 25th May2018, within the first year of INTEGRATE, and it was immediately obvious that the implications of GDPR on PN/CT services must be addressed.

Healthcare providers and community health workers interviewed expressed concerns on the legality of performing of PN services within the new regulation. Article 9, 2 provides a clear provision to allow the continued practice of partner notification for reasons of public health interest if utilizing safeguards to the data subject. In countries that allow healthcare-assisted partner notification, this provision expressly provides them the ability to engage in partner notification [23]. In countries where patient referral is the only method of partner notification supported through legislation, GDPR is not a concern, as patient referral does not involve the healthcare worker sharing any personal data. However, Integrate has demonstrated a clear need to clarify these points with the healthcare and community health workers who perform PN/CT services, as many cited these concerns as preventing them from carrying out their work. It is crucial that any training on PN/CT includes the topic of GDPR in order to alleviate any fears healthcare providers or community health workers may have. At the time of writing there were no studies which discussed or analysed the effects of GDPR on PN/CT services.

## Conclusions

A key finding of the project was the difficulties of incorporating TB contact tracing. Experts highlighted that integrating TB contact tracing with the other infectious diseases included in this project would be difficult and likely unwarranted, both due to the different mode of transmission of TB and the well-established historical pathways for TB in public health systems. However, the success of TB services presents an ideal model to draw from when strengthening other PN systems. Improved training of PN service staff coupled with the introduction of national guidelines for partner notification could greatly improve PN outcomes by empowering staff to support patients. There is a need for increased awareness of the benefits of partner notification, and support from policy makers to adequately resource PN services. Our project identified and established an online repository of country specific guidelines for CT/PN and identified the need for piloted trainings for partner notification in certain countries that were part of our project. Future challenges include a need to understand how different key populations utilize tools and methods of partner notification, how to safely INTEGRATE CT/PN into current legal frameworks, and to engage more EU partners to address the issues of CT/PN.

## Data Availability

Available data can be accessed via Zenodo: 10.5281/zenodo.4518702. National guidelines and documentation were collected from each country, translated and uploaded to a public online repository of documents which can be accessed through https://integrateja.eu/content/partner-notification-guidelines The entire GDPR regulations can be accessed at https://gdpr-info.eu/ The survey report on country requirements for PN/CT can be accessed at www.integrateja.eu

## Abbreviations

PN: Partner Notification
CT: Contact Tracing
TB: Tuberculosis
ECDC: European Centre for Disease Control
WHO: World Health Organization
HIV: Human Immunodeficiency Virus
HBV: Hepatitis B Virus
HCV: Hepatitis C Virus
STI: Sexually Transmitted Infection
SOP: Standard Operating Procedures
IUSTI: International Union against Sexually Transmitted Infections
LTBI: latent TB infection
ICT: Information Communication Technology
EU: European Union
EEA: European Economic Area
ERS: European Respiratory Society
NGO: Non-Government Organization
GDPR: General Data Protection Regulation
STIF: Sexually Transmitted Infections Foundation
BASHH: British Association for Sexual Health and HIV
UNAIDS: United Nations Programme on HIV/AIDS
